# Inequalities in access to water and soap matter for the COVID-19 response in sub-Saharan Africa

**DOI:** 10.1186/s12939-020-01199-z

**Published:** 2020-06-03

**Authors:** Safia S. Jiwani, Daniel A. Antiporta

**Affiliations:** 1 Department of International Health, Johns Hopkins Bloomberg School of Public Health, 615 N Wolfe St, Baltimore, MD 21205 USA; 2 Department of Epidemiology, Johns Hopkins Bloomberg School of Public Health, Baltimore, MD USA

**Keywords:** Inequalities, Hand hygiene, Water and soap, COVID-19, Sub-Saharan Africa

## Abstract

The COVID-19 pandemic has spread rapidly since the first case notification of the WHO in December 2019. Lacking an effective treatment, countries have implemented non-pharmaceutical interventions including social distancing measures and have encouraged maintaining adequate and frequent hand hygiene to slow down the disease transmission. Although access to clean water and soap is universal in high-income settings, it remains a basic need many do not have in low- and middle-income settings.

We analyzed data from Demographic and Health Surveys (DHS) of 16 countries in sub-Saharan Africa, using the most recent survey since 2015. Differences in the percentage of households with an observed handwashing place with water and soap were estimated by place of residence and wealth quintiles. Equiplots showed wide within-country disparities, disproportionately affecting the poorest households and rural residents, who represent the majority of the population in most of the countries.

Social inequalities in access to water and soap matter for the COVID-19 response in sub-Saharan Africa. Interventions such as mass distribution of soap and ensuring access to clean water, along with other preventive strategies should be scaled up to reach the most vulnerable populations.

The COVID-19 pandemic has spread across 185 countries [1] with overwhelming rates of viral transmission. The sub-Saharan African region is believed yet to reach its peak of the epidemic curve, with over 46,000 cases reported as of May 12th [2]. Lacking a safe and effective vaccine, countries have implemented strategies to slow the transmission of disease and prevent the overburdening of their health systems. However, social inequalities in this region challenge an equitable response to the pandemic.

Social distancing measures such as home-stay policies and curfews have been put in place globally. These measures, though necessary, are difficult to implement in a region like sub-Saharan Africa, where more than 40% of people live under 1.9 USD a day [3], and up to two thirds of jobs come from the informal sector [4], precluding many from health insurance and secured income. Overcrowding and informal settlements further exacerbate the feasibility of such mitigation strategies, particularly in a region where the median average household size is 4.8, surpassing 8 in countries such as Senegal and The Gambia [5].

Besides home-stay policies, handwashing with water and soap [6, 7] is one of the most effective interventions, recommended by the WHO and the CDC, to minimize the risk of infection at community-level. While access to water and sanitation is universal in high-income settings, it remains a basic need that many do not have in sub-Saharan Africa.

We analyzed the most recent Demographic and Health Surveys (DHS) of 16 countries in sub-Saharan Africa since 2015. The DHS are nationally-representative household surveys that provide data on health and population indicators [8]. Our main outcome was the proportion of households having water and soap where a place for handwashing was observed. Inequalities were estimated using absolute differences between urban and rural, as well as richest and poorest households, and visualized using equiplots weighted by the population size.

The results from 16 countries in sub-Saharan Africa indicate that, on average, only 33.5% of households with an observed handwashing place at home have water and soap. We found large differences across countries, with national estimates ranging from 5% in Burundi to 64% in Angola, albeit covering different time periods between 2015 and 2018. Rural residents, who represent the majority of the population in the region, have a much lower access compared to their urban counterparts. Urban-rural disparities are wide in all countries, reaching up to 41.8 percentage points in Rwanda, where rural residents represent 82.8% of the total population and only a quarter of them have access to handwashing with water and soap. This basic need remains astonishingly low even in urban areas in countries such as Malawi (16.9%), where the urban-rural gap is narrower. In contrast, Angola presents a different pattern where more than 50% of both urban and rural residents have access to adequate hand hygiene.

Inequalities between the richest and poorest households are as alarming, revealing gaps as large as 63.7 percentage points in South Africa. Burundi had the lowest access regionally, showing 3.8% of rural residents and 1.7% of the poorest households having water and soap at home. Equiplots by place of residence and wealth are shown in Fig. 1. Our findings are based on the households in which a fixed or mobile place used for handwashing was observed, which ranges from 99% in Burundi to 11% in Rwanda. Thus, households that did not have a handwashing place in the dwelling or did not allow the interviewer to observe the facility are not included in our analyses.

**Fig. 1:**
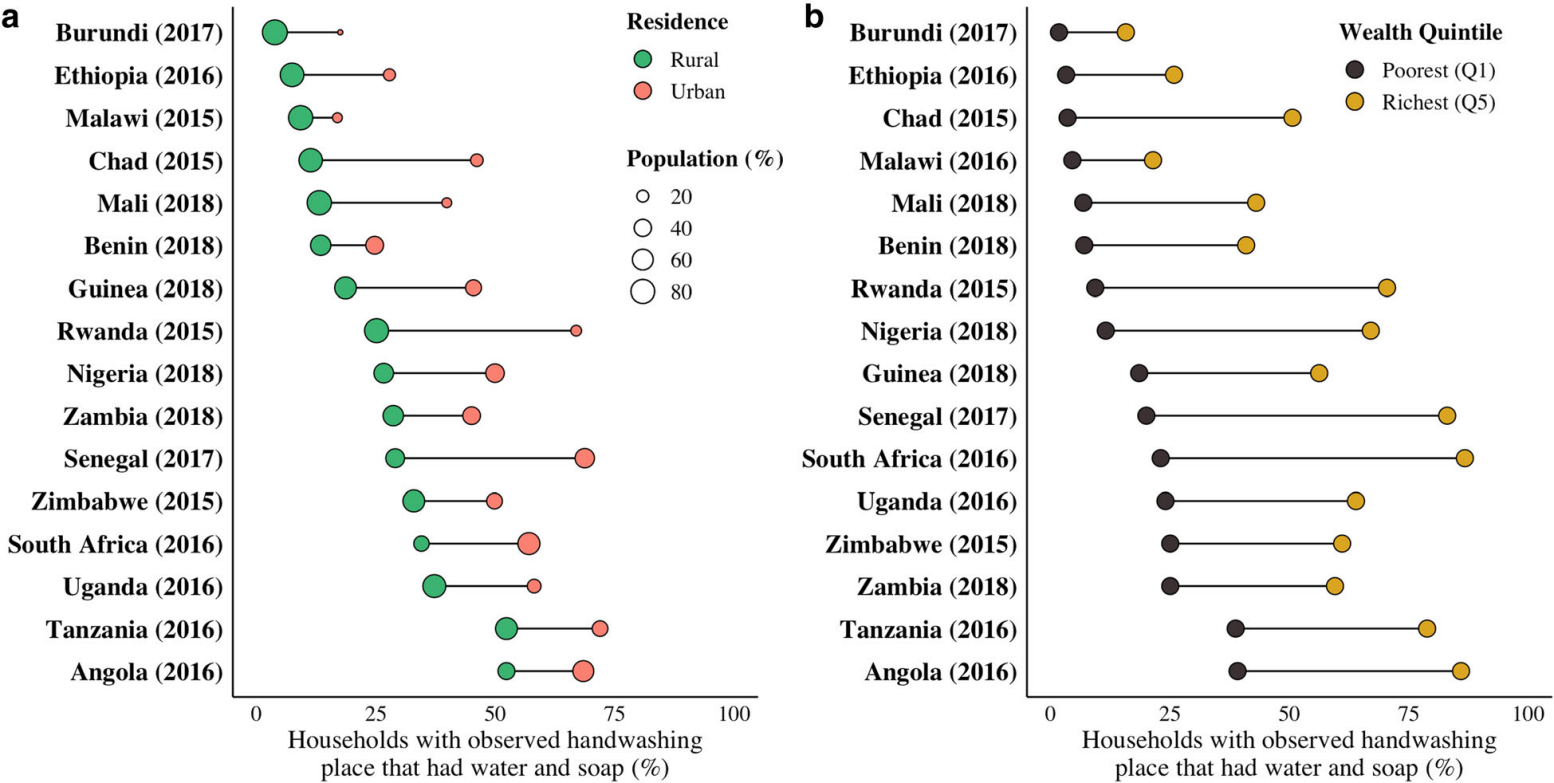
Inequalities in the proportion of households with an observed handwashing place that had water and soap, by place of residence (panel **a**) and wealth (panel **b**)

As the region faces a ravaging and highly contagious virus, many still do not have the basic human right of clean water nor soap for handwashing in their homes. It has never been more urgent to ensure access to essential hand hygiene needs, especially for the rural and poorest who are in a battlefield without this simple, yet powerful, shield against COVID-19. The relevance of access to adequate hand hygiene to prevent the spread of disease ought to be considered by public health officials and scientists for program implementation and disease transmission modelling purposes, respectively.

Action has been taken on this front in several countries in the region where handwashing stations with soap were implemented at public transportation sites in the past months as an early response to the pandemic [9, 10]. Similarly, “tippy taps” have been put in place and are designed to enable safe handwashing specifically in rural areas with no running water [11]. We applaud these strategies and suggest that interventions such as mass distribution of soap and ensuring access to clean water, along with other preventive strategies be widely scaled up, particularly in rural areas and informal settlements.

## Data Availability

The datasets generated and/or analyzed during the current study are available at https://dhsprogram.com/data/available-datasets.cfm or https://www.statcompiler.com/en/
